# Pragmatic randomised clinical trials using electronic health records: general practitioner views on a model of a priori consent

**DOI:** 10.1186/s13063-018-2658-8

**Published:** 2018-05-16

**Authors:** Thomas Hills, Alex Semprini, Richard Beasley

**Affiliations:** Medical Research Institute of New Zealand, Private Bag 7902, Wellington, 6242 New Zealand

**Keywords:** Pragmatic randomised controlled trial, Informed consent, Comparative effectiveness research, Pharmacology, Trial design, Electronic health records

## Abstract

Pragmatic randomised clinical trials could use existing electronic health records (EHRs) to identify trial participants, perform randomisation, and to collect follow-up data. Achieving adequate informed consent in routine care and clinician recruitment have been identified as key barriers to this approach to clinical trials. We propose a model where written informed consent for a pragmatic comparative effectiveness trial is obtained in advance by the research team, recorded in the EHR, and then confirmed by the general practitioner (GP) at the time of enrolment. The EHR software then randomly assigns a patient to one of two treatments. Follow-up data is collected in the EHR. Twenty-two of 23 GPs surveyed (96%) were ‘definitely’ or ‘probably’ comfortable with confirming consent. Twenty-one out of 23 GPs (91%) were ‘definitely’ or ‘probably’ comfortable with a patient being randomised to one of two comparable drugs during a routine consultation. Twenty-two out of 23 GPs (96%) were ‘definitely’ or ‘probably’ comfortable with allowing the electronic system to randomise a patient to drug A or drug B and generate a prescription. Ten out of 23 GPs (43%) identified time constraints as the main hurdle to conducting this sort of research in the primary care setting. On average, it was felt that 6.5 min, in addition to a usual consult, would be acceptable to complete enrolment. Our survey found this model of a comparative effectiveness trial to be acceptable to the majority of GPs.

**Electronic supplementary material:**

The online version of this article (10.1186/s13063-018-2658-8) contains supplementary material, which is available to authorized users.

## Background

Pragmatic randomised clinical trials (RCTs) are emerging as powerful research strategies that seek to generate findings with greater external validity when compared with traditional RCTs. Pragmatic RCTs test the effectiveness of an intervention within the existing health system. Study participants, and the context of their care, reflect treatment in the ‘real world’ with randomised trial treatments, follow-up, and data collection occurring in a wider health system that is authentic to usual practice [1, 2]. Primary care electronic health records (EHRs) are an appealing tool to identify patients for pragmatic RCTs, record participant enrolment, perform randomisation, follow participants over time, and facilitate collection of patient-relevant outcome data. The benefits of this strategy were heralded 5 years ago [3], but very few point-of-care pragmatic RCTs have been conducted using primary care EHRs.

Retropro (ISRCTN33113202) and eLung (ISRCTN72035428) were point-of-care pragmatic RCTs conducted using EHRs in the United Kingdom’s National Health Service. Retropro randomised patients with a high cardiovascular risk profile to either simvastatin or atorvastatin whereas eLung randomised patients with exacerbations of chronic obstructive airways disease to immediate antibiotics, deferred antibiotics, or non-use of antibiotics. A National Institute for Health Research (NIHR) report evaluated the successes and difficulties of these trials and concluded that clinician recruitment was a major challenge [4].

Interview data from general practitioners (GPs) involved in the eLung study identified a lack of time within a routine patient consultation as the over-riding concern with this model for conducting RCTs, particularly the challenge of obtaining and documenting informed consent within a regular consultation [4]. This is in keeping with other work that identified achieving informed consent as a key barrier to pragmatic RCTs in general [5, 6]. Lastly, Good Clinical Practice inspectors stated that: *‘subjects should be given ample time to understand the implications of the study’* and that *‘recruitment at the same consultation as the screening may be less acceptable’* [4].

We propose a modified pragmatic RCT design centred on a priori consent to address these concerns and present feedback from the GPs likely to be involved in future trials.

## Methodology

A convenient sample of GPs, predominantly members of a GP research network associated with the Medical Research Institute, were contacted by email with information on a proposed model for conducting pragmatic RCTs in primary care (see Additional file 1) and a survey on its acceptability (see Additional file 2). Data from the electronic survey was collected and managed using REDCap electronic data capture tools hosted at the Medical Research Institute of New Zealand (MRINZ) [7].

## Results

Twenty-three GPs completed the survey. Twenty-one GPs (91%) were ‘definitely’ or ‘probably’ comfortable with a patient being randomised to one of two comparable drugs during a routine consultation; the remaining two GPs were ‘not sure’ and no GPs were ‘probably not’ or ‘definitely not’ comfortable with this approach.

We proposed a system of a priori consent – where patients are contacted prospectively (before being diagnosed with the relevant condition), provided with access to written information on the trial, able to ask questions directly of the research team, and who then opt in by giving their written informed consent – either for a specific study or for comparative effectiveness trials more generally – before study enrolment. We have recently demonstrated that a priori consent was preferable to patients in this context [8]. A priori consent is then documented in the EHR. At any point during the study, patients can contact the research team to ask questions and to withdraw their consent. If/when a patient becomes eligible (for example, by being diagnosed with the relevant medical condition), the EHR highlights the patient’s eligibility and written consent in the medical record. The GP can confirm with the patient that they consent to entering the trial. Twenty-two GPs (96%) were ‘definitely’ or ‘probably’ comfortable with confirming consent using the electronic record system; the remaining GP was ‘not sure’.

Generally, randomisation to one of two commonly used treatments will occur if patients fulfil the following criteria: they are diagnosed with the relevant medical condition in the EHR, informed consent was given a priori, and this consent to participate has been confirmed by the GP. The EHR software would then randomly assign the patient to one of two treatment arms. Twenty-two GPs (96%) were ‘definitely’ or ‘probably’ comfortable with allowing the electronic system to randomise that patient to drug A or drug B and generate a prescription; the remaining GP was ‘not sure’.

Ten out of 23 GPs (43%) identified time constraints as the primary hurdle to conducting this sort of research in general practice, making it the most commonly identified barrier above practice team participation (2/23, 9%) and software limitations (2/23, 9%). On average, the 23 GPs felt that 6.5 min, in addition to a usual consult, would be acceptable to complete enrolment.

## Conclusions

Conducting pragmatic RCTs that utilise EHR software to randomise patients to one of two or more commonly used treatments was generally an acceptable strategy for this small group of GPs who are likely to be involved in pilot, pragmatic RCT projects. An important limitation of our survey relates to the convenient sampling methodology and these opinions may not reflect those in the wider general practice community. In designing such trials, it is important minimise additional demands on GPs’ time. Shifting the responsibility for achieving written informed consent from the GP to the study team, by obtaining this consent from potentially eligible patients a priori, may render participation in pragmatic RCT research more appealing for GPs.

We plan to undertake pragmatic RCTs in primary care to answer comparative effectiveness questions for commonly prescribed drugs. The choice of study question will need to carefully consider the risks and benefits for patients entering such a pragmatic RCT, as well as the acceptability to GPs and management as important stakeholders in primary care.

## Additional files

Additional file 1: The information that greeted respondents on the landing page of the survey. (PDF 119 kb)

Additional file 2: Survey questions. (PDF 88 kb)

## Abbreviations

EHR: Electronic health record
GP: General practitioner
MRINZ: Medical Research Institute of New Zealand
NIHR: National Institute of Health Research
RCT: Randomised clinical trial

## References

[1] Ford I, Norrie J (2016). Pragmatic trials. N Engl J Med.

[2] Loudon K, Treweek S, Sullivan F, Donnan P, Thorpe KE, Zwarenstein M (2015). The PRECIS-2 tool: designing trials that are fit for purpose. BMJ.

[3] van Staa T-P, Goldacre B, Gulliford M (2012). Pragmatic randomised trials using routine electronic health records: putting them to the test. BMJ.

[4] van Staa T-P, Dyson L, McCann G (2014). The opportunities and challenges of pragmatic point-of-care randomised trials using routinely collected electronic records: evaluations of two exemplar trials. Health Technol Assess.

[5] Pletcher MJ, Lo B, Grady D (2014). Informed consent in randomized quality improvement trials: a critical barrier for learning health systems. JAMA Intern Med.

[6] Faden RR, Beauchamp TL, Kass NE (2014). Informed consent, comparative effectiveness, and learning health care. N Engl J Med.

[7] Harris PA, Taylor R, Thielke R, Payne J, Gonzalez N, Conde JG (2009). Research electronic data capture (REDCap)—A metadata-driven methodology and workflow process for providing translational research informatics support. J Biomed Inform.

[8] Semprini A, Hills T, Braithwaite I, Weatherall M, Beasley R. A priori consent within pragmatic randomised controlled trials: a web-based survey of statin use in primary care. BMJ Innov. 2017;3(4):206. LP-211. Available at: http://innovations.bmj.com/content/3/4/206. Accessed 24 Mar 2018.

