# Supplementary material for: Pragmatic randomised clinical trials using electronic health records: general practitioner views on a model of a priori consent
Source: Trials. 2018 May 16;19:278. doi: 10.1186/s13063-018-2658-8 (PMC5956547; doi:10.1186/s13063-018-2658-8)
Supplement: Supplementary file 1 — The information that greeted respondents on the landing page of the survey. (PDF 119 kb) [file 13063_2018_2658_MOESM1_ESM.pdf]

Additional file 1

This is the information that greeted respondents on the landing page of the survey:

Trial aims and overview

There are many day-to-day treatment decisions where there isn't clear evidence to say which of two (or more) interventions is likely to give a better outcome for patients - particularly in patients in the real world (outside of traditional clinical trials). TIME GP is a novel research strategy that identifies these prescribing decisions during GP consultations, using existing practice software that can trigger enrolment, consent, and randomisation. Data on clinically important outcome measures (e.g. hospitalisation) can then be collected from electronic health records and, as data emerges, information regarding relative efficacy and safety can be obtained. This knowledge is then communicated back to GPs to guide their therapeutic decisions, which are delivered through the medical practice software.

Trial design

The Towards Integrated evidence-based MEdicine in General Practice (TIME GP) research programme proposed here is a novel clinical trial strategy that allows the incorporation of traditional RCT design in routine clinical practice. In this type of trial, patients are randomised to different treatments in a GP setting. It would not involve randomizing patients to experimental therapies or to placebo, but rather treatments commonly used in clinical practice, for which there is uncertainty regarding the preferred choice. On completion of the study the GP software is modified in response to the findings, to ensure patients receive the most beneficial treatment. Utilization of this system would then result in evidence-based practice through allocation of patients to the therapeutic regimen with the best efficacy/safety profile, across the range of medical disorders included in the system. This approach thus has the potential to provide a strategy to answer the comparative efficacy and safety questions that arise for many prescription decisions in day to day practice, and to facilitate the delivery of evidence-based medicine in General Practice.

Informed consent

Crucial to the successful implementation of the TIME GP system is the capability for patients to be aware of the research being undertaken in the practice, similar in some respects to the awareness of medical student teaching by GPs within teaching-based medical centres. Participation in TIME GP would enable medical centres to be designated 'research-based' and 'evidence -medicine based'. Patients within the practices would be invited to provide consent 'in principle' to participate in TIME GP, on the understanding that confirmation of informed consent was obtained during the consultation in which the randomized treatment was prescribed. Patients who provide consent in principle would be regularly advised of the programme of RCTs being undertaken in the medical centre, and provided with information

regarding the studies prior to the consultation in which the randomized treatment is prescribed.

This survey

We consider that the most meaningful way to answer comparative efficacy questions is to run randomised trials in the community, using existing electronic systems that can trigger enrolment, consent, and randomisation. We propose that this method of trial design, and the data it will generate, will result in optimal evidence-based medicine, and translate into better outcomes for your patients. The purpose of this survey is to establish attitudes to this new way of conducting research within the GP network and to brainstorm potential clinical scenarios for future trials.
