# Supplementary material for: Pragmatic randomised clinical trials using electronic health records: general practitioner views on a model of a priori consent
Source: Trials. 2018 May 16;19:278. doi: 10.1186/s13063-018-2658-8 (PMC5956547; doi:10.1186/s13063-018-2658-8)
Supplement: Supplementary file 2 — Survey questions. (PDF 88 kb) [file 13063_2018_2658_MOESM2_ESM.pdf]

Additional file 2

Survey questions:

Introductory information

A consultation results in the diagnosis of a condition for which two drugs (A or B) may be prescribed depending on GP preference, experience etc. A clinical trial is running in your practice involving the randomisation of patients starting treatment to either drug A or B, in order to establish comparative effectiveness, head-to-head. Assume the following and answer the questions below, circle answers where appropriate:

- Ethical and Medsafe approval for the trial.
- Randomisation and consent confirmation are incorporated into the practice management software.

Question one

Would you feel comfortable with a patient being randomised to one of two comparable drugs (A or B) during a routine consultation?
Definitely
Probably
Not sure
Probably not
Definitely not
[choose one option]

Question two

How much time in addition to an average consult would be acceptable to complete enrolment (in minutes)? [free text]

Question three

Assuming a total of an additional 5 minutes per patient (inclusive of any administrative costs), what would be the appropriate level of compensation for your practice (in NZD)?

Further information

Integrating this research into your practice. Clearly the process of entering a patient in a trial must be as efficient as possible and must have minimal impact on the running of the surgery. One potential scenario is as follows: Imagine detailed study information was sent to eligible patients within the practice, patients had the opportunity to discuss the study with the coordinating research team, and then return a signed informed consent form a priori. These patients would then be flagged via the electronic record when the relevant diagnosis was

made. If a patient presented with the condition of interest and the choice between prescribing drug A and B was flagged by your practice software:

Question four

Would you feel comfortable confirming consent using the electronic record system?
Definitely
Probably
Not sure
Probably not
Definitely not
[choose one option]

Question five

Would you feel comfortable allowing the electronic record system to randomise that patient to drug A or B and generate a prescription?
Definitely
Probably
Not sure
Probably not
Definitely not
[choose one option]

The survey then proceeded to ask questions about motivation to engage in this practice, perceived hurdles, and GP suggestions for areas of clinical uncertainty where this trial design might be useful, to help the authors plan pilot studies.
